# Factors Affecting Blood Pressure Variability: Lessons Learned from Two Systematic Reviews of Randomized Controlled Trials

**DOI:** 10.1371/journal.pone.0005673

**Published:** 2009-05-22

**Authors:** Vijaya M. Musini, James M. Wright

**Affiliations:** 1 Department of Anesthesiology, Pharmacology and Therapeutics, Faculty of Medicine, University of British Columbia, Vancouver, British Columbia, Canada; 2 Department of Medicine, Faculty of Medicine, University of British Columbia, Vancouver, British Columbia, Canada; Leiden University Medical Center, Netherlands

## Abstract

Systematic reviews can often reveal much more than the original objective of the work. The objectives of this retrospective analysis were to answer three basic questions about blood pressure variability: 1) Does blood pressure entry criterion have an effect on baseline blood pressure variability? 2) Do thiazide diuretics have a significant effect on blood pressure variability? and 3) Does systolic blood pressure vary to the same degree as diastolic blood pressure? This analysis of blood pressure variability is based on resting standardized research setting BP readings from two systematic reviews evaluating blood pressure lowering efficacy of thiazide diuretics from double blind randomized controlled trials in 33,611 patients with primary hypertension. The standard deviation reported in trials was the focus of the research and the unit of analysis. When a threshold systolic or diastolic blood pressure value is used to determine entry into a trial, baseline variability is significantly decreased, systolic from 14.0 to 9.3 mmHg and diastolic from 8.4 to 5.3 mmHg. Thiazides do not change BP variability as the standard deviation and coefficient of variation of systolic blood pressure and diastolic blood pressure did not differ between thiazide and placebo groups at end of treatment. The coefficient of variation of systolic blood pressure was significantly greater than the coefficient of variation of diastolic blood pressure. Entry criterion decreases the baseline blood pressure variability. Treatment with a thiazide diuretic does not affect blood pressure variability. Systolic blood pressure varies to a greater degree than diastolic blood pressure.

## Introduction

Blood pressure (BP) measurements are highly variable. This is a fact that is commonly not appreciated and variability of blood pressure in an individual could be as important as the magnitude of the blood pressure. Measured blood pressure varies due to a large number of factors such as measurement technique, accuracy of equipment, and multiple patient factors such as anxiety. Even if these factors are controlled, blood pressure is subject to biological variation from beat to beat, minute to minute, and day to day. Each blood pressure measurement is therefore analogous to a single sample from a population of blood pressures. However, it is a patient's mean blood pressure over months and years that are thought to determine his or her risk of cardiovascular disease. In order to increase the precision of the estimated blood pressure, clinical diagnosis is based on the average of 2 to 3 measurements taken after resting for 5 minutes in a non-stimulating environment. Despite such standardized procedures, BP remains highly variable both within and between individuals. However, both this accepted fact and the ease of describing such variability are not well appreciated. Understanding to what extent BP is variable is very important since the large variability of BP impacts diagnosis of hypertension, clinical management of elevated BP and number of drugs prescribed to achieve “BP control”.

BP variability has been shown to increase with increasing blood pressure and correlate with target-organ damage, independent of absolute BP values [1]. However, the importance of BP variability as an independent risk factor remains controversial. In a study by Pierdomenico S et al, after adjustment for other covariates in a Cox multivariate analysis, the adverse prognostic impact of high BP variability was no longer evident [2]. In fact the prognostic value of BP variability has not been tested by proper longitudinal studies and the few available ones are limited by small study size, short follow up or conclusions based on surrogate markers (progression of left ventricular hypertrophy or arterial wall thickening) rather than on the incidence of hard end points, such as cardiovascular events [3]. A better exploration of BP variability and of the influence of drugs on BP variability may improve understanding of the mechanisms involved in BP changes induced by drugs. We know that drugs reduce cardiovascular risks, in different ways and to various extents, by different mechanisms; however what is surprising is that we do not clearly know the impact of these drugs on BP variability.

We have used the availability of a large amount of resting research setting BP data from 33,611 patients, accumulated as part of two systematic reviews, to answer whether thiazides affect BP variability and describe some other characteristics of BP variability [4]–[7].

The objective of this study was to use standard deviation data available from trials meeting the inclusion criteria of two systematic reviews to answer the following three questions about BP variability:

1. Does blood pressure entry criterion have an effect on baseline BP variability?
2. Do thiazide diuretics have a significant effect on BP variability?
3. Does systolic blood pressure (SBP) vary to the same degree as diastolic blood pressure (DBP)?

## Methods

**Figure 1:**
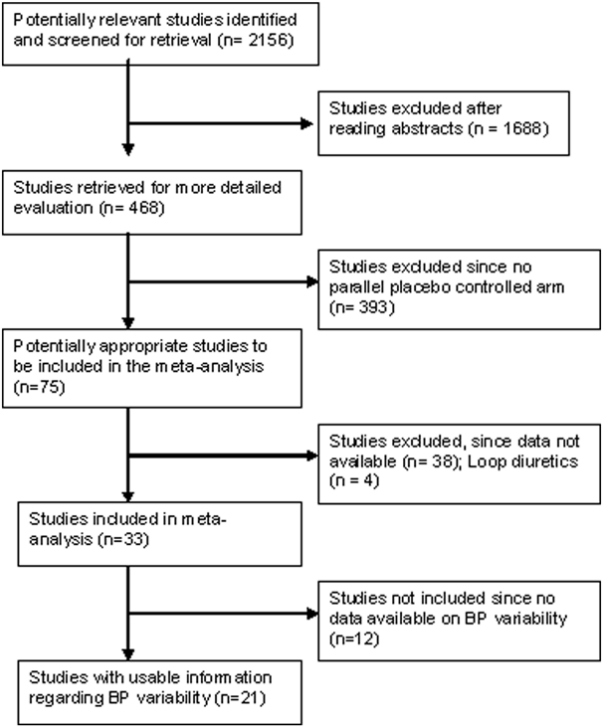
Quorum diagram for systematic review 1.

**Figure 2:**
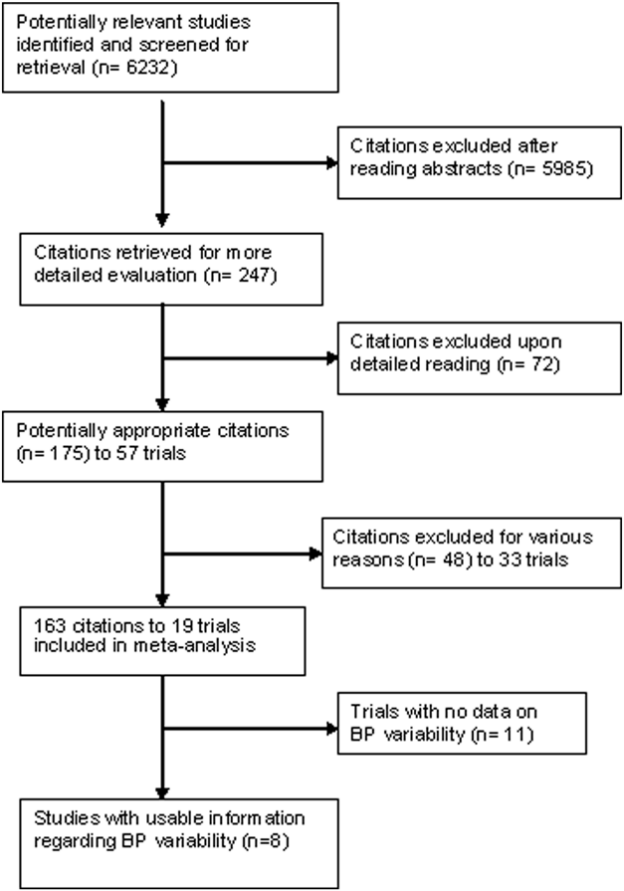
Quorum diagram for systematic review 2.

Standard Cochrane Hypertension Review group search strategies was applied and the following databases were used - Medline, EMBASE, and the Cochrane Clinical Trial Register from 1966–2000 for systematic review 1 and up to 1998 for systematic review 2 to identify trials meeting the inclusion criteria. References of previously published systematic reviews and bibliographic citations of included studies were used to identify any additional trials [5], [7]. Refer Figure 1 and 2 for quorum diagram.

### Inclusion criteria

Both reviews included double blind randomized controlled trials (RCTs) in adult patients with primary hypertension (defined as SBP ≥140 mm Hg and/or DBP ≥90 mm Hg without an identifiable cause). ***Review 1*** –compared thiazide monotherapy with placebo for 3–12 weeks duration and ***Review 2*** – compared thiazide as first-line therapy with placebo or untreated controls for at least one year of therapy [4]–[7]. Although using individual patient data would provide the most information about BP variability, such analysis requires access to the raw data from trials, which is seldom available in published trials. In this analysis we used the standard deviation (SD) estimate of BP variability reported in RCTs as the unit of analysis. In the included trials, blood pressures were resting readings in a standardized research setting: mean of 2 to 3 BP measurements (supine, sitting or standing) taken after 5 minutes rest by trained certified technician or nurse using standardized techniques with a mercury BP manometer. The SBP reading was defined as the reading at the first Korotkoff sound and DBP as the reading at the last Korotkoff sound. Similar methods were used to measure blood pressure at multiple visits during the trial period. Therefore our analysis reflects total variability which includes both the within individual variability and between individual variability of resting BP taken in this standardized research setting. When the mean BP measurements were similar we used the SD at different time periods in each trial as our unit of measure and expressed our results as the mean SD plus or minus standard error (SE). When the mean BP measurement differed we used the coefficient of variation (CV), the mean divided by the SD as the best way of comparing the variability between the two measures.

## Results

A total of 33 trials included in Review 1 (4,811 patients) and 16 trials included in Review 2 (29,351 patients) met the inclusion criteria for the primary objectives of the two systematic reviews [4], [6]. 21/33 trials in Review 1 and 8/19 trials in Review 2 reported the baseline SBP and DBP variability. 21/33 trials in Review 1 and 3/19 trials in review 2 reported the end of treatment SBP and DBP variability.

In review 1 the resting baseline BP (mean of 3 readings) was 159/97 mm Hg and thiazide diuretic monotherapy as compared to placebo reduced SBP by −6.8(−7.7 to −5.9) mmHg and DBP by −4.7(−5.8 to −3.5) mm Hg over a mean duration of 8.8 weeks [4]. In Review 2, the mean baseline BP was 163/95 mmHg and first-line thiazide therapy plus further stepped care drugs as necessary reduced systolic blood pressure by −14.7 (−15.1 to −14.2) mm Hg and diastolic blood pressure by −6.5 (−6.8 to −6.2) mm Hg after one year of treatment [6].

### 1. Does BP entry criterion have an effect on baseline BP variability?

Trial entry criteria affected BP variability at baseline. When a threshold SBP level is part of or the sole entry criterion, baseline SBP variability as assessed by SD is significantly decreased from 14.0 to 9.3 mmHg. (see Table 1 for complete results). Likewise when a threshold DBP level is part of or the sole the entry criterion, baseline DBP variability as assessed by SD is significantly decreased from 8.4 to 5.3 mmHg. (see Table 1).

**Table 1:** Does the blood pressure entry criterion have an effect on the baseline blood pressure variability?

Baseline values (Review 1 and 2)	Entry based on DBP criterion	Entry based on SBP criterion	Entry based on either SBP or DBP criterion
**Mean SD of SBP±SE mmHg**	14.0±0.6 (n = 41)	9.3±1.1* (n = 5)	9.7±1.6* (n = 10)
**Mean SD of DBP±SE mmHg**	5.3±0.3† (n = 44)	8.4±0.7 (n = 4)	4.6±0.6† (n = 10)

SD = Standard Deviation; SE = Standard Error; SBP = Systolic Blood Pressure; DBP = Diastolic Blood Pressure; n = number of observations.

* p<0.01 unpaired t test of mean SD of SBP as compared to entry based on DBP criterion.

† p<0.01 unpaired t test of mean SD of DBP as compared to entry based on SBP criterion.

### 2. Do thiazides diuretics have a significant effect on BP variability?

Only review 1 data could be used to answer this question as in review 2 other drugs besides thiazides were added [4]. As 31/33 trials in this review used DBP ≥90 mmHg as entry criteria and we have demonstrated that this reduced the variability of DBP at baseline (see above), we were mostly limited to SBP variability to answer this question. In Table 2 this is demonstrated in two ways: Firstly variability in SBP did not differ in the thiazide group between baseline and end of treatment (paired t test). Secondly, end of treatment SBP SD did not differ between the placebo and thiazide group using an unpaired t test. We have further verified this by comparing end of treatment diastolic SD using unpaired t test in the placebo and thiazide treated group and they are also not significantly different. (see Table 2). It must be remembered that thiazides reduce the mean systolic and diastolic BP by −6.8/−4.7 mmHg respectively and therefore the mean blood pressures are different at end of treatment. In that circumstance CV is a better way to compare the BP variabilities as it corrects for the difference in mean BP. In this instance the CV for systolic (9.9%) and diastolic (8.2%) in the thiazide group are numerically higher than the respective placebo groups, systolic (9.4%) and diastolic (7.2%), but they are not statistically significantly different.

**Table 2:** Do thiazides diuretics have a significant effect on blood pressure variability?

Review 1	Thiazide group	Placebo group
**SD of SBP±SE mmHg**		
**Baseline**	14.9±0.99 (n = 14)∧	14.1±0.75 (n = 14)∧
**End of treatment** ∧∧	14.3±0.71 (n = 14)	14.0±0.82 (n = 14)
**CV of SBP±SE %**		
**Baseline**	9.5±0.57 (n = 14)∧	9.0±0.39 (n = 14)∧
**End of treatment** ∧∧	9.9±0.42 (n = 14)	9.4±0.53 (n = 14)
**SD of DBP±SE** * **mmHg**		
**End of treatment** ∧∧	7.6±0.37 (n = 23)	6.9±0.39 (n = 23)
**CV of DBP±SE** * **%**		
**End of treatment** ∧∧	8.2±0.39 (n = 23)	7.2±0.43 (n = 23)

SD = Standard Deviation; SE = Standard Error; SBP = Systolic Blood Pressure; DBP = Diastolic Blood Pressure; CV = Coefficient of Variation; n = number of observations.

∧ Paired t test between baseline versus end of treatment is not significant for SD of SBP as well as CV of SBP in both thiazide and placebo groups.

* Only end of treatment values for SD as well as CV of DBP were analyzed since baseline values are confounded.

∧∧ Unpaired t test at end of treatment between thiazide versus placebo groups is not significant for SD as well as CV of both SBP and DBP.

### 3. Does SBP vary to the same degree as the DBP?

For this analysis we used all the unconfounded measures of systolic BP variability and diastolic BP variability from both reviews. Systolic blood pressure SD as expected is greater than diastolic blood pressure SD. Comparing the variability of the two using CV showed the CV of SBP (9.2%) was significantly greater than CV of DBP (8.3%). The results are shown in Table 3.

**Table 3:** Does systolic blood pressure vary to the same degree as diastolic blood pressure?

Review (1+2)	Unconfounded baseline plus end of treatment values in treatment and control groups
**SD of SBP±SE mmHg** ∧	14.0±0.4 (n = 88)
**SD of DBP±SE mmHg**	7.7+0.3 (n = 52)
**CV of SBP±SE %** ∧∧	9.2±0.2 (n = 88)
**CV of DBP±SE %**	8.3+0.4 (n = 52)

SD = Standard Deviation; SE = Standard Error; SBP = Systolic Blood Pressure; DBP = Diastolic Blood Pressure; CV = Coefficient of Variation; n = number of observations.

∧ p<0.0001 unpaired t test SD of SBP versus DBP.

∧∧ p = 0.04 unpaired t test CV of SBP versus DBP.

## Discussion

We have demonstrated in this retrospective analysis that one of the main factors that affect blood pressure variability in the research setting is whether systolic or diastolic BP is used to decide whether the patient is eligible for entry into a trial. When diastolic blood pressure is used, it artificially reduces the variability of diastolic blood pressure at baseline. Similarly, if systolic blood pressure is used, it artificially reduces the variability of systolic blood pressure at baseline. In this assessment the magnitude of this effect in absolute terms is quite large (4.7 mmHg for SBP and 3.1 mmHg for DBP). Some reduction in variability for measures used as entry criteria is not that surprising as the distribution of baseline BP values is truncated at the threshold level of BP required for entry into the trial. However, the large magnitude of the effect suggests that it also reflects clustering of BP measurements at the threshold entry criterion level, an effect that is likely to reflect measurement bias. For example patients with blood pressure slightly below the required entry level might have their measurement increased by the researcher so that they meet the entry level and can be recruited into the trial. The HOT trial, one of the largest hypertension trials, is a good example of this phenomenon [8]. In the HOT trial 18,970 patients were randomized to three different TARGET blood pressures and a DBP of at least 100 mmHg was the entry criteria. In the HOT trial the baseline systolic BP SD was 14.4, which is similar to our estimate of the value 14.0 mmHg noted in Table 1 and 3. However, in the HOT trial the diastolic blood pressure baseline standard deviation was 3.4 mmHg. This is 4.3 mmHg lower than our estimate of diastolic blood pressure SD, 7.7 mmHg (see Table 3). This suggests that in the HOT trial there must have been a large number of patients who had baseline diastolic blood pressures of 100 mmHg thus partially explaining the large reductions in BP seen in that trial in all 3 target groups. Furthermore the end of treatment SD for both systolic, 11.6 mmHg, and diastolic blood pressure, 5.1 mmHg, are less than the expected SD of about 14 mmHg for systolic and 8 mmHg for diastolic. This suggests that either that the drugs used in that trial reduced BP variability or more likely that the process of attempting to achieve a target blood pressure affected the blood pressure measurements reported in a way that decreased their variability. Knowledge of the expected variability of BP can be used, as in this example, to detect data, of questionable validity. When a marked difference in variability in BP from the expected SD of blood pressures demonstrated here is seen in a trial, it could be an indication that the data are fraudulent.

In contrast to the HOT trial the ALLHAT trial randomized 33,357 patients most of whom were selected because they were previously treated [9]. There was therefore no threshold BP that had to be achieved. In the ALLHAT trial the entry SBP SD was 16 mmHg and the DBP SD was 10 mmHg. This is higher than our estimates of BP variability in the research setting and suggests that BP variability in a cohort of treated patients is a little higher than in an untreated population. Since this analysis shows that the magnitude of the mean SBP/DBP reduction due to thiazides, 7/5 mmHg in Review 1 and 15/7 mm Hg in Review 2, is the same as or lower than mean BP variability of 14/10 mmHg, it is clear that clinicians are going to have a very difficult time assessing whether an antihypertensive drug is reducing BP and by what magnitude.

The most important question addressed in this analysis is whether thiazides have an effect on BP variability. Thiazides have been shown to lower blood pressure in many trials, but as far as we are aware nobody has asked or tested whether they have an effect on BP variability. If thiazides (or other antihypertensive treatments) lower or raise BP variability, this could be clinically useful or harmful, respectively. The data we have accumulated suggests that thiazides do not have much effect on BP variability. However, the lack of significance of BP variability between treated and control groups could reflect the absence of any real difference, or lack of power to show a difference. The small increase in both systolic and diastolic CV in the end-of-treatment thiazide group though not statistically significant, makes it probable that thiazides do not decrease BP variability and possible that they could increase BP variability. This should be tested with other databases and preferably databases where individual patient data are available. If there is an increase in variability associated with thiazide treatment it would be important to determine whether it was an increase in interpatient or intrapatient variability. It is particularly the setting of an increase in intrapatient variability that an antihypertensive therapy could increase risk to patients.

Systolic and diastolic BP measurements are dependent on each other; however, there are physiologic settings where systolic BP rises more than diastolic such as during exercise and as muscular arteries lose their elasticity as a part of normal aging. In clinical trials, resting blood pressure is measured in a standardized way. We are not aware of other settings where the variability of systolic and diastolic blood pressure has been directly compared using the coefficient of variation (the mean measurement divided by the SD). In this case we have used all the unconfounded estimates of systolic BP and compared them with all the unconfounded estimates of diastolic BP to increase the chance of showing a difference. In the event the systolic CV was statistically significantly greater than the diastolic CV. This may reflect a true physiological difference. However, it is more likely due to an artifact of the method of measurement. In this case measurements were auscultatory using a mercury manometer. This means that the systolic BP is measured first and the diastolic BP is measured after a short delay. Because of this difference in timing of the two measures patient factors could contribute to the difference in variability e.g. Patients are more relaxed as the pressure in the cuff decreases. Alternatively, it is easier to accurately measure systolic blood pressure (appearance of first Korotkoff sounds) than diastolic blood pressure (disappearance of Korotkoff sounds). Thus the difficulty in detecting the disappearance could lead to a greater likelihood of guessing, which would be expected to artificially lower the variability of diastolic blood pressure. It will be important to repeat this analysis with other data and in other settings. For example a study of blood pressures measured with automatic BP machines using an oscillometric technique may not show a difference in variability of systolic and diastolic blood pressure, thus providing evidence in favor of this being an artificial difference caused by the technique of measurement. Whatever the explanation for the statistically greater variability of systolic blood pressure the magnitude of the increase in variability is small and probably not clinically significant. We do not think that it is a reason to suggest that diastolic blood pressure is a more reliable measure.

In conclusion, systematic reviews can often reveal much more than the original objective of the work. Blood pressure variability as estimated by SD is an important measure and researchers in the area should be familiar with the average magnitude of that variability, 14 mmHg for systolic and 8 mmHg for diastolic, and the factors that can affect it. The impact of the large variability of SBP/DBP needs to be taken in to account when hypertension is diagnosed and when considering what represents “BP control” under treatment.

Most importantly, we need to learn more about the relationship between BP variability and cardiovascular outcomes plus the effect of specific drug treatments on blood pressure variability. We believe that BP variability is a neglected but important measure that deserves more attention.

## References

[1] Parati G (2005). Blood pressure variability: its measurement and significance in hypertension.. Journal of Hypertension.

[2] Pierdomenico SD, Lapenna D, Tommaso RD, Carlo SD, Esposito AL (2006). Blood pressure variability and cardiovascular risk in treated hypertensive patients.. Am J Hypertens.

[3] Mancia G, Bombelli M, Facchetti R, Madotto F, Corraro G (2007). Long-term prognostic value of blood pressure variability in the general population. Results of the pressioni arteriose monitorate e Loro associazioni study.. Hypertension.

[4] Musini VM (2000). A systematic review of the blood pressure lowering efficacy of thiazide diuretics in the treatment of adult patients with primary hypertension..

[5] Musini VM, Wright JM, Jauca CD, Bassett K (2002). Blood pressure lowering efficacy of thiazide diuretics for primary hypertension. Cochrane Database of systematic reviews issue 3. Art No.: CD003824..

[6] Wright JM, Lee C, Chambers KC (1999). Systematic review of antihypertensive therapies: Does the evidence assist in choosing a first-line drug?. Canadian Medical Association Journal.

[7] Wright JM, Musini VM (1999). First-line drugs for hypertension. Cochrane Database of systematic reviews issue 3. Art No.: CD001841..

[8] Hansson L, Zanchetti A, Carruthers SG, Dahlof D, Elmfeldt D (1998). Effects of intensive blood pressure lowering and low dose aspirin in patients with hypertension: principal results of the Hypertension Optimal Treatment (HOT) randomized trial.. Lancet.

[9] The ALLHAT Officers and Coordinators for the ALLHAT Collaborative Research Group (2002). Major outcomes in high-risk hypertensive patients randomized to angiotensin-converting enzyme inhibitor or calcium channel blocker vs diuretic: The Antihypertensive and Lipid-Lowering Treatment to Prevent Heart Attack Trial (ALLHAT).. JAMA.

